# Genetic Similarity of Island Populations of Tent Caterpillars during Successive Outbreaks

**DOI:** 10.1371/journal.pone.0096679

**Published:** 2014-05-23

**Authors:** Michelle T. Franklin, Judith H. Myers, Jenny S. Cory

**Affiliations:** 1 Department of Biological Sciences, Simon Fraser University, Burnaby, BC, Canada; 2 Department of Zoology, and Biodiversity Research Centre, University of British Columbia, Vancouver, BC, Canada; Institut National de la Recherche Agronomique (INRA), France

## Abstract

Cyclic or fluctuating populations experience regular periods of low population density. Genetic bottlenecks during these periods could give rise to temporal or spatial genetic differentiation of populations. High levels of movement among increasing populations, however, could ameliorate any differences and could also synchronize the dynamics of geographically separated populations. We use microsatellite markers to investigate the genetic differentiation of four island and one mainland population of western tent caterpillars, *Malacosoma californicum pluviale*, in two periods of peak or pre-peak density separated by 8 years. Populations showed high levels of genetic variation and little genetic differentiation either temporally between peaks or spatially among sites. Mitochondrial haplotypes were also shared between one island population and one mainland population in the two years studied. An isolation-by-distance analysis showed the F_ST_ values of the two geographically closest populations to have the highest level of differentiation in both years. We conclude that high levels of dispersal among populations maintain both synchrony of population dynamics and override potential genetic differentiation that might occur during population troughs. As far we are aware, this is the first time that genetic similarity between temporally separated population outbreaks in insects has been investigated. A review of genetic data for both vertebrate and invertebrate species of cyclic animals shows that a lack of spatial genetic differentiation is typical, and may result from high levels of dispersal associated with fluctuating dynamics.

## Introduction

The geographic structure and genetic differentiation of populations are determined by demographic processes and dispersal [1]. Of particular interest in this regard are populations that regularly fluctuate in density: cyclic species. These include a number of species of voles and lemmings, snowshoe hares, grouse and some forest Lepidoptera. For these species, population densities can fluctuate over orders of magnitude with peak densities occurring every 3 (small mammals) to 11 (snowshoe hares and forest Lepidoptera) years. An interesting phenomenon associated with cyclic species is that populations often fluctuate synchronously over large geographic areas [2], [3].

Three mechanisms have been proposed as potential causes of the synchronous dynamics among geographically separated populations of cyclic species: (1) sufficient movement of individuals synchronizes populations, (2) widespread weather patterns influence the populations, and/or (3) widespread movement of natural enemies synchronize population declines [4]. Dispersal is most likely to occur in increasing and peak populations and would contribute to the maintenance of synchrony of population dynamics and genetic similarity among them. Mechanisms 2 and 3 would not be as likely to influence genetic structure of populations.

The genetic structure of cyclic populations could be related to their dynamics, in several ways. Elton [5] was likely the first to propose that cyclic population crashes could reduce genetic variation and cause populations to become genetically differentiated. He also pointed out that genetic variability could increase with population growth if selection is relaxed during the phase of population increase. A third possibility is that density-dependent selection or genetic feedback could occur and cause population cycles [6] and predictable modifications of the genetic composition of populations. Much of the work on genetics and population cycles has focused on mammals: voles, lemmings and snowshoe hares [7], [8], [9], [10], [11]. The relationship between genetic change and the cyclic population dynamics of northern mammals has recently been comprehensively reviewed by Norén and Angerbjörn [12]. They conclude that genetic variation tends to be high and that dispersal is sufficient to overwhelm genetic drift in cyclic mammals. Thus dispersal among high-density populations counteracts the potential influence of inbreeding or selection [13], while also increasing the potential for evolution [1] [14]. Some authors, however, suggest that intermediate levels of dispersal allow for the greatest adaptive divergence among populations ([15] and references therein).

Patterns of genetic variation of species of forest Lepidoptera with cyclic population dynamics have received little attention compared to studies of mammals. Social interactions among mammals are likely to influence the occurrence and timing of dispersal over the population cycle and this is important for maintaining high genetic variation and low differentiation [15]. For moths, however, movement is likely to be more passive, and thus for these animals, patterns of genetic variation among populations and years may differ from those of mammals.

**Figure 1:**
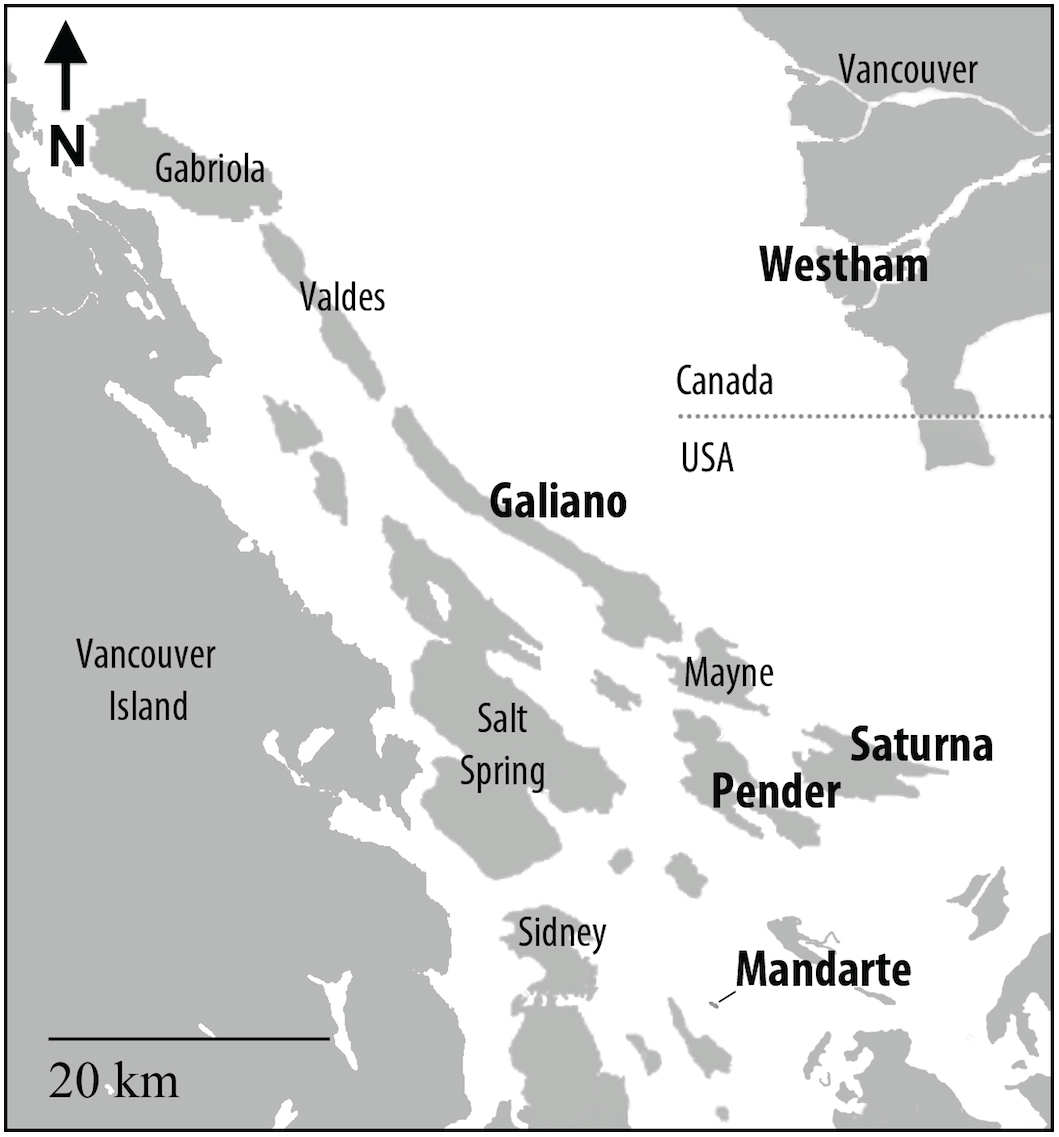
Map of the Southern Gulf Islands and the lower mainland in British Columbia. Islands labeled in bold font were sampled for this study. Arrow indicates north.

**Figure 2:**
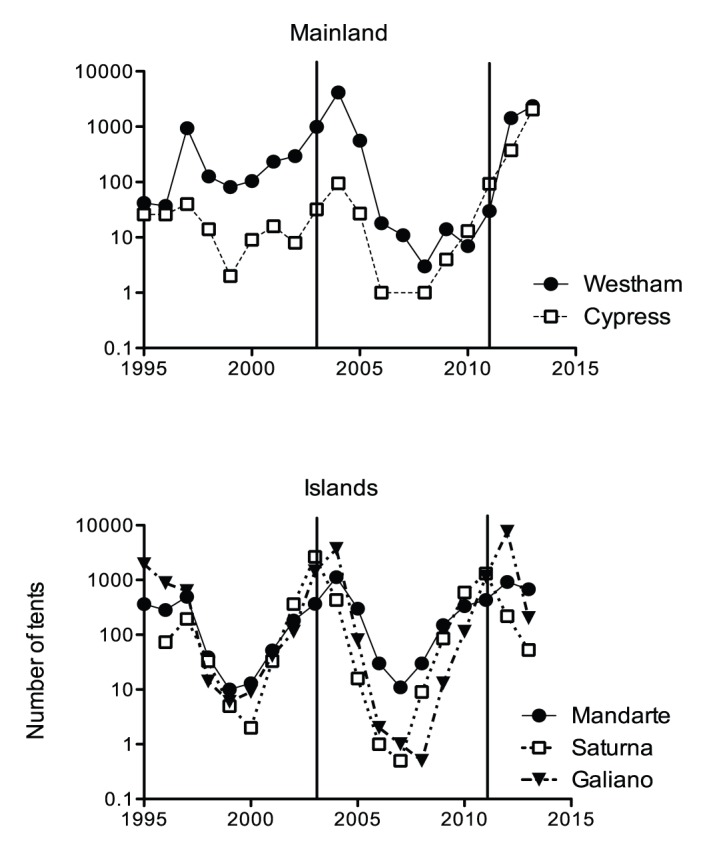
Population trends for western tent caterpillars on three southern Gulf Islands and one mainland site. The Pender Island population was not counted but was observed to be high in 2003 and 2011. Vertical lines indicate the years that were sampled for genetic analysis.

A good example of cyclic dynamics and synchronous outbreaks in an insect is that of western tent caterpillars, *Malacosoma californicum pluviale* (Lepidoptera: Lasiocampidae). Western tent caterpillars occur primarily in western North America from British Columbia to California. They have one generation a year and feed on a variety of deciduous trees. Gregarious larvae form silken tents and live in family groups until the last instar. On the Southern Gulf Islands in British Columbia, Canada (Figure 1), population outbreaks are relatively synchronous and peak population densities are reached within a year of each other approximately every 9 years as shown in Figure 2 ([16], [17] and Myers & Cory, unpublished data). The conspicuous tents facilitate the monitoring of populations even at low density. Tents that represent individual families are very sparse in the troughs of the population fluctuations and populations go locally extinct in some places [2]. Thus genetic bottlenecks are likely to occur on a regular basis. The gregarious behavior of tent caterpillars probably adds to the potential for inbreeding in this species at low densities as well; larvae sometimes pupate in the tent and this possibly results in siblings mating. This could contribute to the severity of the bottleneck and genetic drift.

Here we use variation in microsatellite markers to test the hypothesis that severe bottlenecks and genetic drift during the troughs of population density of western tent caterpillars cause genetic differentiation among populations or between two outbreaks within populations. The alternative hypothesis is that dispersal among populations of western tent caterpillars maintains synchronous dynamics and genetic similarity across peaks and this would be supported if populations were not genetically differentiated. We test these hypotheses by comparing five peak or pre-peak populations of western tent caterpillars in 2003 to those eight years later in 2011 to determine if different genetic patterns emerged following the intervening low density in 2007, if genetic variation is maintained, and if island populations are genetically differentiated.

## Methods

### Field Collections

Late instar tent caterpillar larvae and pupae were collected during the spring and summer of 2003 and 2011 when population sizes were increasing or had reached peak density (Saturna Island), on four islands in southern British Columbia (BC) Canada including: Galiano Island (48° 55′ 19″, −123° 25′ 13″), Mandarte Island (48° 38′ 01″, −123° 17′ 14″), Pender Island (48° 45′ 07″, −123° 13′ 11″) and Saturna Island (48° 46′ 32″, −123° 07′ 55″). The fifth population, which we consider to be a mainland population, is on Westham Island that is separated from the mainland by two arms of the Fraser River (49° 05′ 52″, −123° 10′ 19″) (Figure 1). Larvae and pupae were brought back to the laboratory where the larvae were immediately frozen and pupae reared to adult moths, sexed and then frozen at −20°C or −80°C for later genetic analysis. With the exception of Pender Island, the size of the *M. c. pluviale* population was assessed annually by counting the number of tents in the study area or the whole island in the case of Mandarte Island, which occupies 7 ha. These locations are described in more detail in [16]. Slight variation among the population dynamics occurs (Figure 2). The population on Saturna Island reached peak density in both 2003 and 2011, while populations on Galiano, Mandarte and Pender were at pre-peak density those years. The population at Westham was still relatively low in 2011 when the others were high, but it increased by 2012 in synchrony with another mainland population that we monitored but did not use for genetic analysis, Cypress (Figure 2).

For the isolation by distance analysis we determined the shortest distance between study sites based on their geographic coordinates (latitude and longitude) using Google Map. Since these were island sites, much of the distance between them is over water.

### DNA Extraction and Microsatellite Analysis

DNeasy Blood and Tissue kits (QIAGEN, Ontario, Canada) were used to extract DNA from larvae and moths. DNA was extracted from between 11 and 41 individuals from each of the sites in 2003 and 2011 for a total of 248 individuals. Only one individual was used per tent caterpillar family. Eight microsatellites that had been proven to be highly variable when characterized in a previous study of *M. c. pluviale* were selected for the analysis of population structure [18]. PCR reactions and conditions were as described in [18], with the exception of a reduction in the concentration of MgCl_2_ from 1.0 mM to 0.75 mM to reduce stutter for primers A106 and B101.

PCR products were denatured, loaded on a 6% polyacrylamide gel, and electrophoresis was performed for 1.5 hours on a LICOR 4300 automated sequencer (LICOR Inc., NE, USA). Four size standards (50–350 bp or 50–700 bp LICOR Inc.), negative controls (no DNA) and two reference samples were run on each 64 well gel to ensure that assigned scores were consistent among gels. Gels were scored using SAGA GT version 3.3A for microsatellites (LICOR Inc.).

### Sequencing

To compare haplotypes and genetic similarity between sites we chose two populations for further analysis: Westham Island and Galiano Island. Westham Island lies approximately 30 km to the northeast of Galiano Island across the Strait of Georgia and the two sites are separated by open water. Prevailing westerly winds (data from Environment Canada) could assist moth movement between these locations and thus similar genetic patterns could indicate migration of moths between them.

We sequenced an intergenic spacer region that had previously been found to be highly variable in a number of *Malacosoma* species (up to 154 bp, Felix Sperling, personal communication). We sequenced this region plus part of the COI gene for 12 individuals from Galiano Island in 2003, 9 individuals from Galiano Island in 2011 and 9 from Westham Island in both 2003 and 2011. PCR reactions used 10 ng of genomic DNA, 0.2 mM dNTP, (1x) PCR reaction buffer (Invitrogen), 0.5 units of native Taq (Invitrogen), 2 mM MgCl_2_ (Fermentas, Canada, ON), 5 pmol of forward primer (K698: 5′ TAC AAT TTA TCG CCT AAA CTT CAG CC 3′) and reverse primer (K699: 5′ AGG AGG ATA AAC AGT TCA (C/T)CC 3′) (Eurofins MGW Operon, Huntsville, AL, USA), and 16.4 µl H_2_0 for a total reaction volume of 25 µl. PCR conditions were as follows: 94°C for 2 min, cycled 35 times at 94°C for 30 s, 45°C for 1 min, and 72°C for 30 s, with a final extension of 72°C for 5 min. Products were purified using sodium acetate precipitation and sent to Eurofins MGW Operon (AL, USA) for sequencing. Bioedit Sequence Alignment Editor [19] was used to align sequences and a BLASTn search was performed to identify significantly similar sequences. Sequences have been deposited in the GenBank database (GenBank Nos. KF956114–KF956152). We used TCS version 1.21 [20] to construct a haplotype network based on a combined analysis of the intergenic spacer region and partial COI.

### Genetic Diversity

The number of observed alleles (Na), number of effective alleles (Ae), allele frequency, expected heterozygosity (He), and observed heterozygosity (Ho) were estimated using GenoDive version 2.0b22 [21] for each population in 2003 and 2011. Mean rarefied allelic richness (R_T_) was calculated in FSTAT version 2.9.3 for each population in the two sampling years [22].

### Population Structure

FSTAT was used to estimate global F_ST_ values for all populations sampled and separately for the two sampling years. Pairwise F_ST_ values were calculated in Genodive and significance was assessed using 100,000 permutations. Bonferroni correction was applied to correct for multiple comparisons. An isolation-by-distance analysis was carried out using standardized F_ST_ values and log distance between sites using GenAIEx 6.5 [23]. The Bayesian clustering program STRUCTURE 2.3.3 [24] was used to examine the spatial and temporal structure of *M. c. pluviale* populations. First, simulations were run including all eight microsatellite loci for the 248 individuals surveyed from the five populations in both 2003 and 2011. Following this, separate analyses were performed for each of the survey years. These analyses included 124 individuals in 2003 and 124 individuals in 2011. We followed the recommendations of [25] when selecting parameter settings. For each cluster (*K* values) from 1 to 10 we ran 20 replicate simulations using a burn-in length of 100,000 and 500,000 Markov chain Monte Carlo (MCMC) repetitions. These parameter settings were appropriate given the convergence of all summary statistics (α, *F, D,* and the likelihood). Simulations were run using the admixture model with correlated allele frequencies. To detect the true number of clusters we calculated the ad hoc statistic, Δ*K* [26]. STRUCTURE HARVESTER [27] and CLUMPP [28] were used to combine data from replicate runs from the same *K* value and DISTRUCT [29] was used to visualize the results.

We used TEMPOFs [30] to estimate the variance effective population size (NeV) for each of the island populations using sampling plan II, where sampled individuals are permanently removed from the population prior to reproduction. This method requires at least two samples from a population to estimate the genetic drift and effective population size between the two dates and reduces any bias that might result from small samples sizes or skewed allele distribution (at the cost of larger standard deviations). The generation time is one year for *M. c. pluviale* and therefore eight generations elapsed between sampling periods. In addition, we have compared these data to two other methods which use the temporal approach to estimate genetic drift and Ne: the likelihood method developed by Wang [31], [32] assuming a migration rate of zero and the Nei & Taijma [32] estimator Fc, using MLNE [33]. We jointly estimated effective population size and migration using MLNE [33] for each island population by defining the source population based on the pooled allele frequencies for all other island populations [34].

## Results

A total of 105 alleles were observed for the eight microsatellites tested. The number of alleles per locus ranged from 4 (B121) to 32 (A106) with an average of 13.12 alleles per locus for the eight loci. Seven of the F_IS_ values for each locus for the five islands and two years were significant after Bonferroni correction (Critical *P* = 0.05/80 = 0.0006; Table 1). An overall significant excess of heterozygotes is indicated by the F_IS_ value (F_IS_ = 0.07, *P*<0.001). This is due to significant departures from Hardy-Weinberg equilibrium observed at three of the loci (B108, B121, and B6) (Table 1). These loci do not appear to be influential in other analyses and removal of these loci from F_ST_ estimates did not significantly change the results.

**Table 1:** F_IS_ values for each microsatellite locus and location combination to test for deviations from Hardy-Weinberg equilibrium.

	Galiano		Mandarte		Pender		Saturna		Westham		All Locations
	2003	2011	2003	2011	2003	2011	2003	2011	2003	2011	
**A106**	−0.042	−0.011	−0.019	0.065	0.111	0.014	0.147	0.048	0.105	0.084	−0.053
**A113**	0.000	−0.070	−0.062	−0.020	−0.133	0.104	−0.110	−0.029	−0.055	−0.011	−0.076
**A117**	0.006	−0.011	−0.004	−0.013	−0.052	0.133	−0.026	0.000	−0.028	−0.087	−0.051
**B101**	−0.021	0.161	−0.032	0.049	−0.047	−0.043	0.035	−0.011	−0.015	0.281	0.009
**B103**	−0.193	−0.095	−0.049	0.133	0.327	−0.007	−0.011	0.176	0.011	0.043	−0.043
**B108**	0.466	0.680*	0.902*	0.685*	0.468	0.349	0.434	0.543	0.419*	0.303	0.484*
**B121**	0.327	0.244	−0.050	0.204	0.650*	0.566*	0.450*	0.608	0.309	0.429	0.329*
**B6**	0.247	0.129	0.125	0.129	0.080	0.315	0.235	−0.154	0.222	0.067	0.107*
**All** **Loci**	0.081	0.120*	0.080	0.141*	0.145	0.146*	0.135*	0.117	0.112*	0.135	0.070*

*Deviations from Hardy-Weinberg equilibrium at a significance level of *P* = 0.05. Bonferroni correction was applied to account for multiple comparisons.

### Sequence Analysis

**Figure 3:**
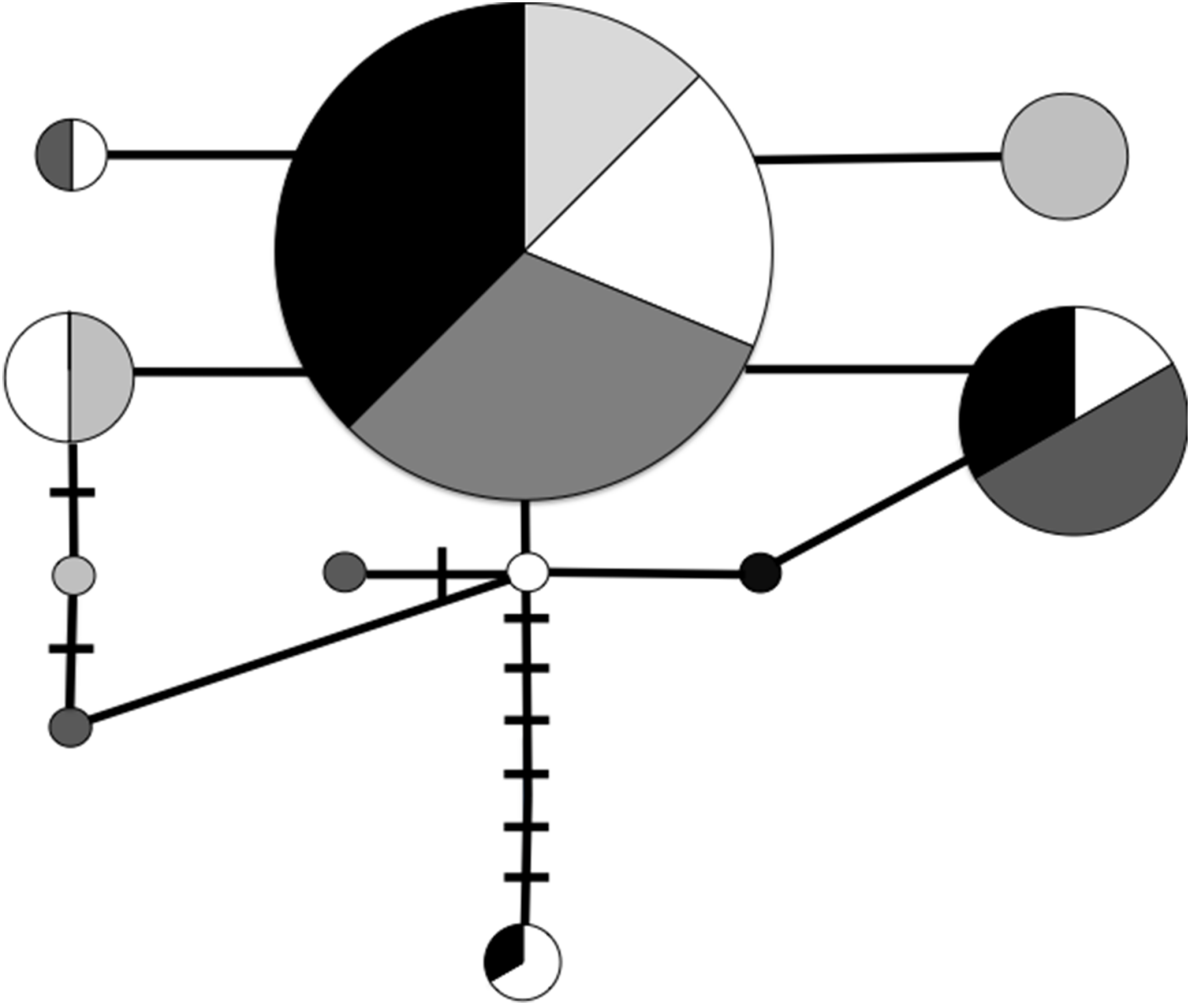
Network of 11 mitochondrial haplotypes of western tent caterpillars from Galiano and Westham Islands based on the intergenic spacer region and partial sequences of cytochrome oxidase I. Individuals from Galiano Island 2003 and 2011 are represented by light gray and white and those from Westham Island 2003 and 2011 are represented by dark gray and black, respectively. The size of each circle is proportional to the number of individuals. Lines connecting each haplotype correspond to a single nucleotide change and black lines represent a single unobserved nucleotide change between haplotypes inferred from parsimony analysis.

Analysis of the intergenic spacer region and partial CO1 revealed 11 distinct haplotypes for Galiano and Westham populations (Figure 3). Although several of the less common haplotypes originated from either Galiano or Westham, there was no clear separation of the two sites and the majority of individuals were grouped within one dominant haplotype that combined both sites and years.

### Genetic Diversity and Population Structure

The number of alleles observed on each island varied from 6.25 to 9.75 over both years and after correction for sample size the effective number of alleles ranged from 4.54 to 6.33 (Table 2). Expected and observed heterozygosity ranged from 0.75 to 0.81 and 0.64 to 0.71 for populations sampled in 2003 and 2011, respectively. None of the diversity measures showed obvious or consistent temporal change between the survey years.

**Table 2:** Genetic diversity estimates for *Malacosoma californicum pluviale* populations from five islands in British Columbia, Canada.

Island	Year	N_a_	A_e_	R_T_	H_O_	H_E_
Galiano	2003	6.25	4.54	5.98	0.69	0.80
	2011	7.88	5.31	6.10	0.70	0.76
Mandarte	2003	9.75	6.33	6.55	0.71	0.81
	2011	7.5	4.95	5.86	0.70	0.76
Pender	2003	9.13	5.16	6.03	0.64	0.75
	2011	8.13	5.04	6.06	0.65	0.76
Saturna	2003	9.25	5.51	6.27	0.64	0.75
	2011	8.13	4.96	5.93	0.66	0.77
Westham	2003	7.00	4.84	6.13	0.68	0.77
	2011	8.63	4.99	6.00	0.67	0.76

N_a_ number of alleles, A_e_ effective number of alleles, R_t_ rarefied allelic richness, H_o_ observed heterozygosity, H_e_ expected heterogyzosity.

**Figure 4:**
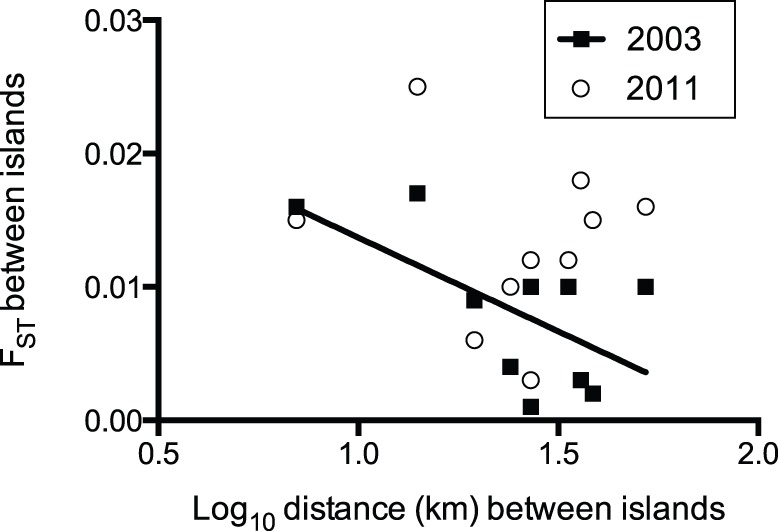
Isolation by distance analysis for western tent caterpillar populations in 2003 and 2011.

The global F_ST_ value for all populations sampled from the five islands in 2003 and 2011 was 0.009, which was significantly different from 0 at *P*<0.001. This indicated a very low level of structure among populations. When examined separately the pairwise F_ST_ values for populations surveyed in 2003 were lower than those in 2011 (F_ST_ 2003 = 0.007, *P<*0.05; F_ST_ 2011 = 0.011, *P<*0.001), although none of the pairwise F_ST_ values were significantly different after Bonferroni correction was applied (Table 3). In 2003 five of the F_ST_ values were less than 0.01 and three of these involved the Pender population and three the Westham population. In 2011 only two pairwise F_ST_ values were less than 0.01: one involving Pender and the other involving Westham. Isolation by distance analysis showed slight significance in 2003 (*P* = 0.04), with the counter intuitive result of F_ST_ decreasing (becoming more similar) with increasing distance. No significant pattern occurred in 2011 (*P* = 0.40) (Figure 4).

**Table 3:** Pairwise F_ST_ comparisons for *Malacosoma pluviale californicum* populations surveyed in 2003 and 2011.

	Gal03	Gal11	Mand03	Mand11	Pend03	Pend11	Sat03	Sat11	West03	West11
**Gal03**	-									
**Gal11**	0.010	-								
**Mand03**	0.010	0.016	-							
**Mand11**	0.012	0.012	0.012	-						
**Pend03**	0.004	0.006	0.009	0.000	-					
**Pend11**	0.005	0.010	0.013	0.006	0.014	-				
**Sat03**	0.010	0.002	0.017	0.018	0.016	0.005	-			
**Sat11**	0.017	0.012	−0.001	0.026	0.018	0.015	0.018	-		
**West03**	−0.001	0.005	0.010	0.008	−0.002	0.005	0.003	0.005	-	
**West11**	0.007	0.003	0.025	0.016	0.007	0.015	−0.002	0.018	0.000	-

Collections were performed in 2003 and 2011 from Galiano (Gal), Mandarte (Mand), Pender (Pend), Saturna (Sat), and Westham Island (West) on the west coast of British Columbia, Canada. One hundred thousand permutations of the data were used to test for significant differences.

In agreement with F_ST_ results, the clustering method implemented in structure identified little population structure when all populations from 2003 and 2011 were analyzed together. Ln Pr(*X|K*) was highest for the model with *K* = 1 indicating there is no spatial or temporal structure among *M. c. pluviale* populations (Likelihood values: K = 1, −7085, K = 2, −7123, K = 3, −7378). Based on the Evanno method, Δ*K* was highest for *K* = 3, however inspection of the bar plot and individual assignment probabilities (*q-hat*) indicated a low probability of individual membership into each cluster for *K* = 3 and lack of structure for this *K* value. Separate examination of populations surveyed in 2003 (Likelihood values: K = 1, −3367, K = 2, −3570, K = 3, −3555.6) and 2011 (Likelihood values: K = 1, −3697, K = 2, −3931.5, K = 3, −4094.6) indicated that Δ*K* was largest for *K* = 2. However, the individuals had a low probability of membership into each of the two clusters (cluster 1: 0.50, cluster 2: 0.50) and supported the grouping of all individuals into a single cluster in both survey years.

### Effective Population Size

Estimates of effective population size for each island population based on Jorde & Ryman’s [30] Fs ranged from 75 to 187, with the Pender Island population having the lowest Ne and Galiano Island the highest (Table 4). The corresponding upper limits for Galiano and Westham Island were infinity indicating that estimates of genetic drift could not be differentiated from that arising from random sampling error. This occurs when estimates for effective population size are low [30]. Fc estimates are within the range of values found with other methods, with the likelihood estimate with no migration being higher than the others. However, likelihood estimates of Ne were low when assuming migration (Ne = 25–50) and indicated a high level of migration among all island populations (m = 0.9–1.0). This is in agreement with F_ST_ values and the STRUCTURE results that indicated a lack of genetic differentiation among island populations.

**Table 4:** Estimates of effective population size (Ne) and migration rate (m) of *Malacosoma californicum pluviale* populations based on temporal samples from surveys in 2003 and 2011.

Island	Ne assuming m = 0			Ne assuming m>0	
	F_s_(Jorde & Ryman 2007)	Likelihood(Wang 2001)	F_c_(Nei & Tajima 1981)	Ne	m
Galiano	187 (77−∞)	357 (155–13053)	176	28 (16–95)	0.9 (0.22−>1)
Mandarte	132 (77–470)	453 (169−>10000)	180	39 (24–131)	1.0 (0.18–>1)
Pender	75 (41–399)	376 (155−>10000)	198	50 (28–121)	1.0 (0.33–>1)
Saturna	84 (53–91)	285 (94−>10000)	124	25 (15–64)	1.0 (0.26–>1)
Westham	112 (49−∞)	256 (78−>10000)	133	31 (16–332)	0.96 (0.09–>1)

## Discussion

Estimates of the genetic structure of four island and a mainland western tent caterpillar populations indicate low levels of genetic differentiation among these populations over scales of tens of kilometers. In addition to spatial similarity, population peaks 8 years apart did not differ. This conclusion is based on the genetic similarity of pre-peak populations as indicated by STRUCTURE, low F_ST_ values, the low likelihood estimates of Ne, and the low diversity of haplotypes shared between populations. The isolation by distance analysis did indicate a weak relationship in 2003 and curiously this was the opposite to the anticipated pattern; closer populations were more genetically differentiated. There is no obvious explanation for this and, given that the result was not repeated in 2011, further data need to be gathered to assess whether this is a consistent pattern. However, it is interesting that the two closest populations have almost exactly the same F_ST_ value in both 2003 and 2011.

It is likely that the lack of genetic differentiation is related to movement of moths among populations including from the islands to the mainland. This suggestion is supported by several lines of evidence. First we know that moth dispersal can reestablish “sink” populations of tent caterpillars when source populations are high [2], [35]. In addition, moth movement of a congener, the forest tent caterpillar, *Malacosoma disstria* was observed by Brown [36] to occur over 450 km from an outbreak area. Thus movement of tent caterpillar moths over tens of kilometers is feasible. The very high rate of population increase in the Westham population between 2011 and 2012 (Figure 2) is compatible with an influx of immigrant moths laying eggs there. In addition, as an example of immigration we observed a new egg mass at an upper elevation, mainland population in July 2012 when the local population was still in the larval stages (Myers and Cory, pers obs.). This can only be explained by an immigrant moth laying the eggs. Finally the typical westerly winds in the evenings and nights when moths are flying in the summer in southwestern BC (Environment Canada Weather records) would potentially assist movement of moths from the island populations towards the mainland. Winds could also facilitate movement of moths among islands. The southern Gulf Islands are in the rain shadow of the coastal mountains of Vancouver Island and the sunnier and dryer conditions there are ideal for tent caterpillars.

In addition to the high levels of genetic similarity among tent caterpillar populations, their population dynamics also show similar temporal patterns. Tent caterpillar populations cycle over four orders of magnitude and can become locally extinct. The resulting population bottlenecks could possibly allow for genetic drift to occur and influence the ability of populations to adapt and evolve. It could also result in genetic differentiation among populations if movements were restricted. However, given the rapid population increase over three to four generations following the trough of population density, genetic diversity should be reestablished [37].

Estimates of effective population size were less than 200 with the use of two of the methods and could not be accurately estimated for two of the populations. We found in an earlier study that multiple paternity was likely to have occurred in a high proportion of families from high-density populations [18]. Waite and Parker [38] showed that multiple paternity increases the variance in male reproductive success and could influence the estimate of Ne.

A lack of genetic differentiation appears to be typical of outbreak and cyclic species of forest Lepidoptera. Eastern tent caterpillars, *M. americanum* populations were shown with electrophoretic markers to have high genetic homogeneity among widely separated populations [39]. Similarly, a genetic analysis of a well-studied cyclic forest lepidopteran, the Larch budmoth, *Zeiraphera diniana*, in Switzerland using allozymes showed a low F_ST_ of 0.002 among larch feeding populations [40]. The genetic structure of the winter moth, *Operophtera brumata*, was studied in Belgium using allozymes [41] and there was no isolation by distance or genetic differentiation on the scale of 10 to 40 km. Winter moth were also studied in the Orkney Isles of Scotland where populations had become established in the last 20 to 30 years due to range expansion [42]. Genetic differentiation based on AFLP variation among populations on three islands was low which suggests high gene flow. The population dynamics of this species has been well studied in Fennoscandia where cyclic waves of expansion occur every 10 years from the east to the west over 3000 km [43]. Ballooning of first instar larvae is thought to explain the high migration of this species in which females are wingless [42]. A review of other studies by Leggett et al. [42] showed that four species of Lepidoptera that are known to have high dispersal had low F_ST_ estimates while four species with low dispersal had higher F_ST_ estimates. An example of a species with strong geographical structure based on AFLP and mitochondrial markers, and one that does not show cyclic dynamics, is pine processionary moth, *Thaumetopoea pityocampa* [44]. The distribution of this pest of pine trees has expanded with warming temperatures and pine cultivation. Population differentiation is thought to reflect this spread and contrasts the results with winter moth [42]. Another noncyclic, lepidopteran species the cabbage looper, *Trichoplusia ni*, migrates annually to British Columbia from permanent populations in southern North America. A high genetic connectivity occurred over the migration route, but some genetic differentiation occurred among seasonal, green house populations in British Columbia [45].

Comparisons of the genetic structure of outbreak and non-outbreak populations have also been carried out in another insect order, the Orthoptera. Outbreak populations of the migratory locust, *Locusta migratoria*, showed much greater genetic homogeneity than did non-outbreaking populations [46]. Similarly the plague locust, *Chortoicetes terminifera* in Australia is characterized by outbreak dynamics and showed no genetic structure across the country [47].

Other non-insect cyclic species also have been shown to lack genetic differentiation even though populations periodically decline to low density. Burton et al. [7] using eight microsatellite markers (the same number as we investigate here) estimated levels of genetic variation (13.4 alleles), expected heterozygotes (0.67) and F_ST_ (0.015) for snowshoe hares in the northern Boreal Forest of Alaska and Yukon. These values are similar to those we report here for cyclic tent caterpillars. They concluded that hares have large evolutionary effective populations. Berthier et al. [9] showed that cyclic populations of water voles, *Arvicola terrestris*, maintain high genetic diversity through migration of increasing populations that wipes out the genetic drift among demes that occurs in low-density populations. Similarly Ehrich and Jorde [8] found low genetic differentiation and high gene flow using mtDNA markers in a study of five species and 72 populations of lemmings in the genera of *Lemmus* and *Dicrostonyx*. They suggest that population fluctuations possibly destabilize the spatial organization and favor the success of migrants. Four-fold variation in mean density of root voles (*Microtus oeconomus*) over six years revealed no change in genetic heterogeneity [11]. Rikalainen et al. [10] used 23 microsatellite loci to study the genetic structure of bank vole, *Myodes glareolus,* populations over eight consecutive years of population fluctuation. They found relatively high levels of genetic heterogeneity but a lack of genetic differentiation among populations at the scale of 10 to 40 km during that time. No signature of population bottlenecks was apparent.

Of particular interest with the western tent caterpillars is the potential for strong selection associated with the epizootic of a nucleopolyhedrovirus that occurs at peak densities which could potentially select for different resistant genotypes during sequential outbreaks [17]. The variation in disease resistance among populations in 2003 is not reflected in the lack of population differentiation seen with the microsatellites. It is, however, unlikely that neutral markers would show a strong signal of changes in other potentially genetically based traits. A similar situation was found for cyclic populations of water voles, *Arvicola terrestris* [9]. Neutral microsatellite markers indicated low population subdivision and low F_ST_ at high densities. Genes associated with the major histocompatibility complex that is related to immune function showed evidence of balancing selection and geographic differentiation among populations at low density. At high density, genetic homogenization was greater than could be explained by drift and migration alone and therefore could have been associated with a general pattern of selection across all populations.

Genetic variation associated with dispersal of geographically separated populations is best studied in metapopulations of the Glanville fritillary butterfly, *Melitaea cinxia* ([48] and citations therein). By using functional genomics tools, Wheat et al. [48] were able to demonstrate variation between new and old populations for loci related to flight and metabolic traits. A more genome wide approach such as this will be required to determine if ecologically relevant, genetic variation occurs among cyclic populations of tent caterpillars.

We conclude that movement of moths from outbreak populations of western tent caterpillars is sufficient to make populations genetically similar and to synchronize their dynamics. This pattern is typical of mammals with cyclic population dynamics and suggests that although opportunities for drift and selection may cause populations to differentiate during low density, movement among populations at outbreak density eliminates that differentiation. Further work is required to determine if genetic differentiation occurs at low density of tent caterpillars, but the lower-density population at Westham in 2011 was similar to the other populations in that year which indicates that this is not the case. Finally, the similar patterns of genetic homogeneity among populations of a variety of cyclic species supports the hypothesis that dispersal is a common attribute of cyclic animal populations and that this maintains genetic similarity and synchronous dynamics over wide geographic ranges.

## Supporting Information

Dataset S1 Microsatellite data for the five *Malacosoma californicum pluviale* populations.
(XLSX)

## References

[1] Slatkin M (1987) Gene flow and the geographic structure of natural populations. Science 236: 787–792. 3576198 10.1126/science.3576198

[2] Myers JH, Cory JS (2013) Population cycles in forest Lepidoptera revisited. Ann Rev Ecol Evol and Syst 44: 565–592.

[3] Krebs CJ, Kielland K, Bryant J, O’Donoghue M, Doyle F, et al (2013) Synchrony in the snowshoe hare (*Lepus americanus*) cycle in northwestern North America, Can J Zool. 91: 562–572.

[4] Liebhold AM, Haynes KJ, Bjornstad ON (2012) Spatial synchrony of insect outbreaks. In: Barbosa P, Letourneau DK, Anurag AA, editors. Insect Outbreaks Revisited. Chichester UK: John Wiley & Sons Ltd. 114–125.

[5] Elton CS (1924) Periodic fluctuations in the numbers of animals: Their causes and effects. Exp Biol 2: 119–163.

[6] Chitty D (1971) The natural selection of self-regulatory behavior in animal populations. In: McLaren IA, editor. Natural Regulation of Animal Populations: Lieber-Atherton. 136–170.

[7] Burton C, Krebs CJ, Taylor EB (2002) Population genetic structure of the cyclic snowshoe hare (*Lepus americanus*) in southwestern Yukon, Canada. Mol Ecol 11: 1689–1701. 12207720 10.1046/j.1365-294x.2002.01566.x

[8] Ehrich D, Jorde PE (2005) High genetic variability despite high-amplitude population cycles in lemmings. J Mammol 86: 380–385.

[9] Berthier K, Charbonnel N, Galan Y, Chaval Y, Cosson J-F (2006) Migration and recovery of the genetic diversity during the increasing phase in cyclic vole populations. Mol Ecol 15: 2665–2676. 16842435 10.1111/j.1365-294X.2006.02959.x

[10] Rikalainen K, Aspi J, Galarza JA, Koskela E, Mappes T (2012) Maintenance of genetic diversity in cyclic populations - a longitudinal analysis in *Myodes glareolus* . Ecol Evol 2: 1491–1502. 22957157 10.1002/ece3.277 PMC3434924

[11] Pilot M, Dabrowski MJ, Jancewicz E, Schtickzelle N, Gliwicz J (2010) Temporally stable genetic variability and dynamic kinship structure in a fluctuating population of the root vole *Microtus oeconomus* . Mol Ecol 19: 2800–2812. 20561198 10.1111/j.1365-294X.2010.04692.x

[12] Norén K, Angerbjörn (2013) Genetic perspectives on northern population cycles: bridging the gap between theory and empirical studies. Biol Rev. 10.1111/brv.12070 24779519

[13] Ingvarsson PK, Whitlock MC (2000) Heterosis increases the effective migration rate. Proc Roy Soc 267: 1321–1326. 10.1098/rspb.2000.1145 PMC1690676 10972127

[14] Ebert D, Haag C, Kirkpatrick M, Riek M, Hottinger JW, et al (2002) A selective advantage to immigrant genes in a *Daphnia* metapopulation. Science 295: 485–488. 11799241 10.1126/science.1067485

[15] Garant D, Forde SE, Hendry AP (2007) The multifarious effect of dispersal and gene flow on contemporary adaptation. Funct Ecol 21: 434–443.

[16] Myers JH (2000) Population fluctuations of western tent caterpillars in southwestern British Columbia. Pop Ecol 42: 231–241.

[17] Cory JS, Myers JH (2009) Within and between population variation in disease resistance in cyclic populations of western tent caterpillars: a test of the disease defence hypothesis. J Anim Ecol 78: 646–655. 19220564 10.1111/j.1365-2656.2008.01519.x

[18] Franklin MT, Ritland CE, Myers JH, Cory JS (2012) Multiple mating and family structure of the western tent caterpillar, *Malacosoma californicum pluviale*: Impact on disease resistance. Plos One 7(5): e37472 doi:10.1371/journal.pone.0037472 22655050 10.1371/journal.pone.0037472 PMC3360058

[19] Hall TA (1999) BioEdit: a user-friendly biological sequence alignment editor and analysis program for Windows 95/98/NT. Nucleic Acids Symp Ser (Oxf) No 41: 95–98.

[20] Clement M, Posada D, Crandll K (2000) TSC: a computer program to estimate gene genealogies. Mol Ecol 9: 1657–1660. 11050560 10.1046/j.1365-294x.2000.01020.x

[21] Meirmans PG, Van Tienderen PH (2004) genotype and genodive: two programs for the analysis of genetic diversity of asexual organisms. Mol Ecol Notes 4: 792–794.

[22] Goudet J (1995) FSTAT (Version 1.2): A Computer Program to Calculate F-Statistics. J Hered 86: 485–486.

[23] Peakall R, Smouse P (2012) GenAIEx 6.5: Genetic analysis in Excel. Population genetic software for teaching and research - an update. Bioinform 28: 2537–2539. 10.1093/bioinformatics/bts460 PMC3463245 22820204

[24] Pritchard JK, Stephens M, Donnelly P (2000) Inference of population structure using multilocus genotype data. Genet 155: 945–959. 10.1093/genetics/155.2.945 PMC1461096 10835412

[25] Gilbert KJ, Andrew RL, Bock DG, Franklin MT, Kane NC, et al (2012) Recommendations for utilizing and reporting population genetic analyses: the reproducibility of genetic clustering using the program structure. Mol Ecol 21: 4925–4930. 22998190 10.1111/j.1365-294X.2012.05754.x

[26] Evanno G, Regnaut S, Goudet J (2005) Detecting the number of clusters of individuals using the software structure: a simulation study. Mol Ecol 14: 2611–2620. 15969739 10.1111/j.1365-294X.2005.02553.x

[27] Earl D, vonHoldt B (2012) STRUCTURE HARVESTER: a website and program for visualizing STRUCTURE output and implementing the Evanno method. Cons Genet Res 4: 359–361.

[28] Jakobsson M, Rosenberg NA (2007) CLUMPP: CLUster matching permutation program version 1.1.2. 10.1093/bioinformatics/btm233 17485429

[29] Rosenberg NA (2004) Distruct: A program for the graphica display of population structure. Mol Ecol Notes 4: 137–138.

[30] Jorde PE, Ryman N (2007) Unbiased estimator for genetic drift and effective population size. Genet 177: 927–935. 10.1534/genetics.107.075481 PMC2034655 17720927

[31] Wang J (2001) A pseudo-likelihood method for estimating effective population size from temporally spaced samples. Genet Res 78: 243–257. 11865714 10.1017/s0016672301005286

[32] Nei M, Tajima F (1981) Genetic drift and estimation of effective population size. Genet 98: 625–640. 10.1093/genetics/98.3.625 PMC1214463 17249104

[33] Wang J, Whitlock MC (2003) Estimating effective population size and migration rates from genetic samples over space and time. Genet 163: 429–446. 10.1093/genetics/163.1.429 PMC1462406 12586728

[34] Larsson LC, Laikre L, André C, Dahlgren TG, Ryman N (2010) Temporally stable genetic structure of heavily exploited Atlantic herring (*Clupea harengus*) in Swedish waters. Heredity 104: 40–51. 19654606 10.1038/hdy.2009.98

[35] Wellington WG (1960) Qualitative changes in natural populations during changes in abundance. C J Zool 38: 289–314.

[36] Brown CE (1965) Mass transport of forest tent caterpillar moths, *Malacosoma disstria*, by a cold front. Can Entomol 97: 1073–1075.

[37] Nei M, Maruyama T, Chakraborty R (1975) Bottleneck effect and genetic variability in populations. Evolution 29: 1–10. 28563291 10.1111/j.1558-5646.1975.tb00807.x

[38] Waite TA, Parker PG (1997) Extra-pair paternity and the effective size of socially monogamous populations. Evolution 51: 620–621. 28565338 10.1111/j.1558-5646.1997.tb02450.x

[39] Costa JT, Ross KG (1994) Hierarchial genetic structure and gene flow in macrogeographic populations of the eastern tent caterpillar (*Malacosoma americanum*). Evolution 48: 1158–1167. 28564470 10.1111/j.1558-5646.1994.tb05302.x

[40] Emelianov I, Mallet J, Baltensweiler W (1995) Genetic differentiation in the larch budmoth *Zeiraphera diniana* (Lepidoptera: Tortricidae): polymorphism, host races or sibling specis? Heredity 75: 416–424.

[41] van Dongen S, Backeljau T, Matthysen E, Dhondt AA (1998) Genetic population structure of the winter moth (*Operophtera brumata* L.) (Lepidoptera, Geometridae) in a fragmented landscape. Heredity 80: 92–100.

[42] Leggett HC, Jones EO, Burke T, Hails RS, Sait SM, et al (2011) Population genetic structure of the winter moth, *Operophtera brumata* Linnaeus, in the Orkney Isles suggests long distance dispersal. Ecol Entomol 36: 318–325.

[43] Tenow O, Nilssen AC, Bylund H, Pettersson R, Battisti A, et al (2012) Geometrid outbreak waves travel across Europe. J Anim Ecol 82: 84–95. 22897224 10.1111/j.1365-2656.2012.02023.x

[44] Salvato P, Battisti A, Concato S, Masutti L, Patarnello T, et al (2002) Genetic differentiation in the winter pine processionary moth (*Thaumetopoea pityocampa-wilkinsoni* complex), inferred by AFLP and mitochondrial DNA markers. Mol Ecol 11: 2435–2444. 12406253 10.1046/j.1365-294x.2002.01631.x

[45] Franklin MT, Ritland CE, Myers JH (2010) Spatial and temporal changes in genetic structure of greenhouse and field populations of cabbage looper, *Trichoplusia ni* . Mol Ecol 19: 1122–1133. 20163547 10.1111/j.1365-294X.2010.04548.x

[46] Chapuis M-P, Loiseau A, Michalakis Y, Lecoq M, Franc A, et al (2009) Outbreaks, gene flow and effective population size in the migratory locust, *Locusta migratoria:* a regional-scale comparative survey. Mol Ecol 18: 792–800. 19207256 10.1111/j.1365-294X.2008.04072.x

[47] Chapuis M-P, Popple J-AM, Berthier K, Simpson SJ, Deveson E, et al (2011) Challenges to assessing connectivity between massive populations of the Australian plague locust. Proc Roy Soc 278: 3152–3160. 10.1098/rspb.2010.2605 PMC3158929 21389030

[48] Wheat CR, Fescemyer HW, Kvist J, Tas E, Verba JC, et al (2011) Functional genomics of life history variation in a butterfly metapopulation. Mol Ecol 20: 1813–1828. 21410806 10.1111/j.1365-294X.2011.05062.x

